# γδ T Cells at the Crossroads of Tuberculosis and COPD: From Early Immunity to Tissue Remodeling

**DOI:** 10.3390/ijms27156965

**Published:** 2026-08-03

**Authors:** Dmitry Oskin, Stanislav Kotlyarov

**Affiliations:** 1 Department of Infectious Diseases and Phthisiology, Ryazan State Medical University, 390026 Ryazan, Russia; 2 Department of Nursing, Ryazan State Medical University, 390026 Ryazan, Russia; skmr1@yandex.ru

**Keywords:** tuberculosis, COPD, gamma delta T cells, Vγ9Vδ2 T cells, macrophages, epithelial barrier, BCG, trained immunity, IL-17

## Abstract

Tuberculosis (TB) and chronic obstructive pulmonary disease (COPD) remain global public health challenges, yet the mechanisms underlying their comorbidity remain poorly understood. COPD is associated with an increased risk of active TB, but the cellular and molecular basis of this association remains unclear. A growing body of evidence suggests that TB is not limited to an intramacrophage infection but is accompanied by a disruption of the local immune balance in lung tissue. This review analyzes the early interface of host–*Mycobacterium tuberculosis* (Mtb) interaction—from pattern recognition signaling pathways and metabolic reprogramming of macrophages to epithelial barrier responses and the delayed αβ T cell response. This review evaluates γδ T cells, particularly phosphoantigen (pAg)-reactive Vγ9Vδ2 cells, as a candidate early integrative component of the host response, positioned between macrophage infection, epithelial stress, mycobacterial antigens, and bacille Calmette–Guérin (BCG)-induced trained immunity. Available evidence suggests that COPD-associated immune alterations may impair mucociliary clearance, antimicrobial peptide production, phagocytosis, and innate T cell responses while favoring cytotoxic reactions, the IL-17A–G-CSF–neutrophil axis, and protease-mediated tissue damage; however, direct mechanistic evidence in patients with TB–COPD remains limited. It is important to note the differences in data obtained from studies in humans, primates, mice, and in vitro: the Vγ9Vδ2 system has no direct analog in laboratory mice, which represents a key limitation for preclinical studies. A key unresolved question remains the nature and functional consequences of γδ T cell changes in patients with coexisting COPD and TB infection. The answer to this question could inform new strategies for the prevention and treatment of TB in patients with COPD, including BCG revaccination, pAg-mediated immunomodulation, and the development of biomarkers to distinguish between protective and harmful early immune responses.

## 1. Introduction

Tuberculosis (TB) remains one of the leading causes of death from a single infectious agent: in 2024 an estimated 10.7 million people fell ill with TB and 1.23 million died of it, and TB once again caused more deaths than any other single pathogen [1]. Its causative agent, *Mycobacterium tuberculosis* (Mtb), has traditionally been regarded primarily as an intracellular pathogen of macrophages. This view is partly justified: macrophages are among the first cells to take up mycobacteria and provide an early intracellular niche for Mtb within the lung. However, the outcome of infection—elimination of the pathogen, latent infection, or active disease—is determined neither by a single cell type nor by a single antimicrobial mechanism. Mtb enters a complex tissue environment in which alveolar and interstitial macrophages, airway and alveolar epithelial cells, tissue-resident T cells, unconventional innate-like T cells—including γδ T cells, mucosal-associated invariant T (MAIT) cells, and invariant natural killer T (iNKT) cells—and local inflammatory mediators operate simultaneously [2].

TB is considered not merely as a macrophage-restricted infection but as a disturbance of the molecular and cellular equilibrium of lung tissue. We focus on molecularly defined host–mycobacteria interfaces, including macrophage pattern recognition pathways, epithelial stress signaling, phosphoantigen (pAg) sensing through the BTN3A1/BTN2A1–Vγ9Vδ2 axis, and bacille Calmette–Guérin (BCG)-induced trained immunity. Almost every component of this response has a dual role. Macrophages are intended to destroy Mtb, yet when phagocytosis and intracellular killing are incomplete, they become a site of mycobacterial persistence [3]. The epithelium restricts pathogen entry, but under certain conditions it may participate in Mtb translocation and persistence [4]. T cells are required for durable control of infection, but an excessive or improperly localized response can damage lung tissue [2]. Thus, the fate of Mtb infection is determined not by the absolute strength of a single protective reaction but by the balance between restriction of bacterial replication and inflammation-induced tissue injury.

γδ T cells occupy a special position in this system. They are not the principal or universal mechanism of anti-TB defense, but they can function as an early integrative compartment between innate recognition and the delayed antigen-specific αβ T cell response. Unlike classical αβ T cells, which migrate to the site of infection only after a considerable delay, γδ T—cells can respond rapidly to microbial metabolites, infected-cell stress signals, and prior BCG vaccination. In humans and non-human primates, pAg-responsive Vγ9Vδ2/Vγ2Vδ2 cells are of particular interest because their activation links mycobacterial metabolism, butyrophilin-dependent sensing, cytokine production, and early macrophage activation. Data from non-human primates indicate that targeted activation of Vγ2Vδ2 cells can enhance control of TB infection [5]. γδ T cells may therefore be viewed as an early molecular bridge connecting macrophage infection, epithelial stress, mycobacterial antigens, BCG-induced trained immunity, and subsequent tissue T cell responses.

This equilibrium is particularly vulnerable in COPD. Tobacco smoke exposure is consistently associated with an increased risk of TB [6,7], and COPD itself has been associated with an increased risk of active TB in population-based data [8]. Recent cohort data suggest that pulmonary TB during the developmental period may contribute to COPD and pre-COPD later in life [9]. COPD should therefore not be considered a passive comorbid diagnosis. Rather, it represents a remodeled lung immune niche in which barrier integrity, macrophage phagocytosis and efferocytosis, epithelial antimicrobial responses, and T cell-mediated inflammation are altered. However, how COPD reshapes the γδ T cell compartment specifically during Mtb infection remains poorly defined.

The reverse arm of this association has a plausible mechanistic basis. Necrotizing Mtb-driven inflammation and cavitation expose the lung to sustained neutrophil- and macrophage-derived proteolysis; matrix metalloproteinases and neutrophil elastase degrade the extracellular matrix. MMP-1 concentrations are raised in the sputum and bronchoalveolar lavage fluid of patients with TB, Mtb selectively upregulates MMP1 expression and secretion in primary human monocytes, and transgenic expression of human MMP-1 is sufficient to produce alveolar destruction and collagen breakdown in Mtb-infected mice, which do not otherwise reproduce human tissue destruction [10]. The fibrotic repair that follows distorts the small airways. Loss of alveolar attachments, bronchiectatic remodeling, incomplete epithelial regeneration and persistent local inflammation can together produce fixed airflow obstruction and impaired gas exchange after microbiological cure [11]. A recent meta-analysis of 32 observational studies quantifies both directions of the relationship: a history of TB was associated with an approximately 2.5-fold higher risk of COPD (pooled OR 2.46, 95% CI 1.95–3.10) and COPD with an approximately 2.2-fold higher risk of TB (pooled OR 2.21, 95% CI 1.57–3.11) [12]. These processes provide a biologically plausible pathway from pulmonary TB to post-TB airflow obstruction; longitudinal data that separate newly developed obstruction from pre-existing COPD, however, remain limited, and the reported prevalence of post-TB lung function impairment varies widely with the population studied and the spirometric criteria applied [13].

The aim of this review is to synthesize current understanding of the early molecular and cellular mechanisms of anti-mycobacterial defense in lung tissue and to examine how these mechanisms may be remodeled in COPD. Particular attention is given to the early host–Mtb interface before the formation of a full αβ T cell response, the position of γδ T cells at the intersection of macrophage infection, epithelial stress signaling, mycobacterial antigen recognition, and BCG-induced immune reprogramming, and the mechanisms by which COPD may shift anti-mycobacterial immunity from early containment toward pathogen persistence, chronic inflammation, and tissue damage. The review also identifies unresolved questions that remain central to the COPD–γδ T cell–Mtb triad.

### Literature Search and Selection Strategy

This work is a narrative mechanistic review and is not a systematic or scoping review; accordingly, the formal PRISMA protocol was not applied. Sources were selected in PubMed/MEDLINE using combinations of terms relating to TB, COPD, γδ T cells, Vγ9Vδ2, macrophages, epithelial cells, BCG, trained immunity, and mycobacterial antigens. Priority was given to studies in humans, work in non-human primates, mechanistic experimental studies, current reviews, and publications that clarify model-specific limitations. Particular attention was paid to distinguishing data obtained in humans, primates, murine models, and in vitro systems. The selection of sources focused on the early cellular response and on the role of γδ T cells and is not intended as an exhaustive survey of the entire literature on the immunology of TB.

PubMed/MEDLINE was used as the primary index because it provides broad and well-indexed coverage of the immunological and mycobacteriological literature on which this review draws, and because its MeSH indexing allowed the mechanistic concepts of interest to be retrieved reliably. To reduce the risk that relevant work indexed elsewhere was missed, the searches were supplemented by backward and forward citation tracking of the key primary studies and reviews, and by hand-searching the reference lists of recent reviews of TB immunology and of COPD pathobiology. The search was last updated in July 2026; no date restriction was applied, and retrieved records were restricted to publications in English. We nonetheless regard reliance on a single bibliographic index as a limitation. Because the review is narrative rather than systematic, no protocol was registered, no formal risk-of-bias appraisal was undertaken, and the selection of sources inevitably reflects the authors’ judgment of mechanistic relevance. To make that judgment auditable, statements in the text are accompanied throughout by an explicit indication of the model system and the level of evidence on which they rest (Box 1).

The principal molecular mechanisms that connect the cellular tiers discussed below are summarized in Box 2.

Box 1 **Hierarchy of Evidence and Model Limitations.**

*Key assumptions when extrapolating data to humans*

**Levels of evidence (in decreasing order of translatability to humans)**

**Human studies—**direct clinical and translational data**Non-human primates—**models with high biological proximity for a number of immune mechanisms**Murine models—**useful for testing mechanisms but require cautious extrapolation**in vitro/ex vivo—**allow a mechanism to be isolated but do not reproduce the entire tissue context
**Principal limitations of interpretation**

**Vγ9Vδ2/BTN3A1–BTN2A1—**the pAg-reactive system is characteristic of humans and primates; standard laboratory mice have no direct functional counterpart.**Granulysin—**participates in the direct anti-mycobacterial activity of cytotoxic cells in humans and primates; absent in ordinary mice.**Intravenous BCG—**pronounced protection has been shown in macaques, but this route of administration cannot be transferred directly to clinical practice.**COPD → γδ T cells → Mtb—**direct data are insufficient; the emphysema model with the IL-17A → G-CSF → neutrophil axis is not equivalent to clinical COPD in TB.**M1/M2 polarization—**a convenient but simplified scheme; single-cell sequencing data reveal a spectrum of coexisting macrophage states.

Box 2 **Key Molecular Mechanisms Discussed in This Review.**

*Host–mycobacteria molecular axes that connect the sections of this review*

**Pattern recognition signaling—**TLR2, NOD2, C-type lectins (mannose receptor, DC-SIGN, Mincle/MCL, Dectin-2), cGAS–STING, and the NLRP3 inflammasome.**Phagosome subversion—**arrest of phagosome maturation and ESX-1-mediated phagosomal membrane damage.**Macrophage immunometabolism—**the glycolytic (Warburg-like) switch, foamy-macrophage formation, and the apoptosis-versus-necrosis decision.**Phosphoantigen sensing—**HMBPP/IPP through the BTN3A1/BTN2A1–Vγ9Vδ2 axis.**γδ T cell effectors—**IFN-γ/TNF activation of macrophages and granulysin-mediated anti-mycobacterial activity.**Damage axes in COPD—**the IL-17A–G-CSF–neutrophil axis and protease-driven injury (neutrophil elastase, MMP-12, NETs).**BCG-induced trained immunity—**NOD2-dependent epigenetic and metabolic reprogramming of myeloid and innate-like cells.

## 2. The Cellular Architecture of Mtb Interaction with Lung Tissue

The lungs are in constant contact with aerosolized antigens and microorganisms, so the local immune response must be rapid enough to contain infection yet restrained enough not to disrupt respiratory function. The encounter of lung tissue with Mtb is not a linear reaction of a single cell but a sequential interaction of several cellular tiers.

The first tier of this interaction is the barrier and early (pre-immune) phagocytosis. The epithelium of the airways and alveoli, together with the alveolar macrophage, primarily determines what fraction of the pathogen reaches the tissue microenvironment at all and how Mtb behaves in the first hours and days after infection. The alveolar macrophage typically becomes the first host cell and the early point of entry for Mtb, whereas interstitial and recruited monocyte-derived macrophages serve as the subsequent site of more active replication and dissemination of the pathogen [14,15,16,17,18]. The epithelium, in turn, acts not as a passive barrier but as an active participant in the early response—and, at the same time, as a possible route for the establishment of Mtb in the tissue [4,19,20,21,22,23,24].

The second tier is the T cell response, which is separated in time. Antigen-specific αβ T cells provide durable control of infection but are engaged with a delay and depend on their localization within lung tissue [2,25,26,27]. Between early innate recognition and the late αβ response lie the rapid unconventional T cells. Among these rapid unconventional T cell populations, γδ T cells are particularly relevant here, especially the pAg-reactive Vγ9Vδ2 subset in humans and non-human primates, which can produce IFN-γ, TNF, and IL-17 within hours [5,28,29,30,31,32]. MAIT cells, iNKT cells, and CD1-restricted T cells belong to the same early-response tier and respond to microbial metabolites and lipids [33,34,35,36,37,38]. In COPD, innate-like T cell compartments may be numerically and functionally impaired [39].

The third tier comprises the cells that set the inflammatory background and its outcome. Neutrophils are able to restrict Mtb early but, on excessive activation, become a source of protease-mediated damage and tissue necrosis [40,41]; dendritic cells link early recognition to T cell activation in the lymph nodes [15]; innate lymphoid cells provide a rapid cytokine response [42]; and B cells and tertiary lymphoid structures (TLS/iBALT) organize the local response but, in chronic inflammation, sustain tissue remodeling in both TB and COPD [43,44].

**Figure 1:**
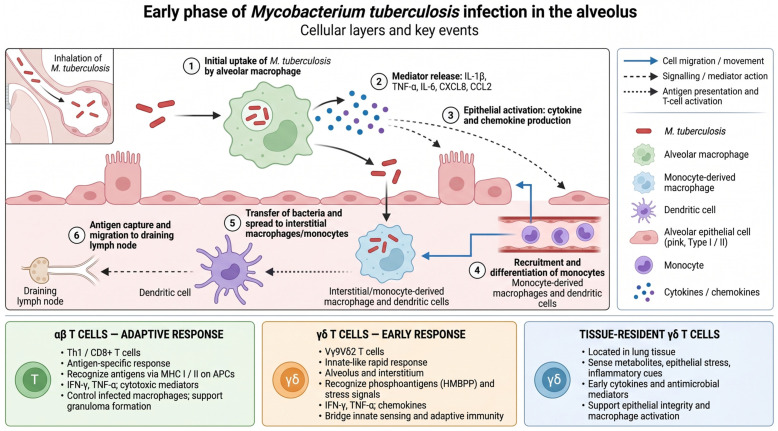
The early phase of *Mycobacterium tuberculosis* (Mtb) infection in the alveolus. Schematic of the alveolus: the alveolar macrophage as the first point of entry for Mtb within the surfactant layer; AT2/AT1 cells and antimicrobial peptides; the transition of infection to interstitial and monocyte-derived macrophages; early engagement of γδ T cells at barrier surfaces; and the delayed influx of αβ T cells from the draining lymph node. Arrows denote the temporal sequence of events rather than causal exclusivity. Created in BioRender. Oskin, D. (2026) https://BioRender.com/uk18e1f (accessed on 31 July 2026).

Thus, in COPD and after prior TB, the early cellular architecture of anti-mycobacterial defense appears to shift from coordinated containment toward a state in which barrier, phagocytic, and innate-like protective mechanisms are weakened, whereas cytotoxic, neutrophilic, and tissue-remodeling responses are amplified. This early pulmonary host–Mtb interface is illustrated in Figure 1, while Table 1 summarizes the protective roles, pathological shifts, and COPD-related relevance of the major cellular populations involved.

## 3. The Macrophage and Mtb: Recognition and the Intracellular Fate of the Bacterium and the Infected Cell

The macrophage is the first to make contact with Mtb. In doing so, a cell whose purpose is to destroy the pathogen often becomes the site of its persistence. The early outcome of infection depends not only on whether Mtb is taken up, but also on the route by which it enters the cell, how the phagosome behaves, which metabolic program the macrophage adopts, and the manner in which the infected cell dies [2,3,16,17,18,49,123,124,125,126,127,128,129].

Recognition of the foreign agent at the first step is set by the structure of the mycobacterial cell wall, which is rich in lipids and glycolipids (ManLAM, mycolic acids, the cord factor TDM, lipoproteins). These molecules serve as ligands for innate immune receptors of various classes—lectin receptors (the mannose receptor, DC-SIGN, Mincle/MCL, Dectin-2), complement and scavenger receptors, as well as Toll-like receptors (TLRs); recognition then continues inside the cell as well (NOD2, the cGAS → STING pathway when mycobacterial DNA reaches the cytosol, and the NLRP3 inflammasome) [2,3,123,130,131,132,133,134,135]. What matters is not the completeness of this list but the fact that the route of entry sets the first branch point: uptake via the mannose receptor and DC-SIGN is accompanied by milder inflammation and may favor the survival of Mtb, whereas recognition of TDM via Mincle/MCL engages the pro-inflammatory Syk → CARD9 pathway, and prolonged signaling through TLR2 can suppress IFN-γ-dependent activation and antigen presentation [2,130,134,135].

After uptake, Mtb resides in a phagosome whose maturation it blocks, keeping the internal environment in an inactive state and preventing its acidification and fusion with lysosomes; cell wall lipids (ManLAM) and secreted phosphatases (PtpA) participate in this. In parallel, the ESX-1 system damages the phagosomal membrane, and part of the bacterial material escapes into the cytosol, activating the cGAS–STING pathway and autophagy (including xenophagy involving ATG5/ATG7 and p62). Autophagy can partially restore control over intracellular Mtb, but the pathogen counteracts this as well, which makes the intracellular conflict protracted and unstable [3,124,125,126].

How Mtb antagonizes autophagy has become clearer, and the mechanisms act at more than one step. The secreted acid phosphatase SapM hydrolyzes phosphatidylinositol 3-phosphate on the phagosomal membrane and thereby impedes phagosome maturation; more recent work places it further upstream still, showing that SapM localizes to lysosomes, dephosphorylates Raptor at Ser792 and so sustains mTORC1 activity, which suppresses the initiation of autophagy rather than only its completion [136]. The protein kinase PknG acts at the opposite end of the pathway: it binds the pleckstrin homology domain of AKT, phosphorylates TBC1D4 and blocks GTP hydrolysis on RAB14, so that autophagosomes form but fail to mature and autophagic flux is interrupted [137]. Both effectors therefore convert autophagy from a bactericidal route into a stalled compartment in which the bacterium persists. The relative weight of these mechanisms in human alveolar macrophages is not established—the evidence comes largely from murine macrophages and cell lines—and whether they are accentuated in the metabolically and functionally altered macrophages of COPD has not been tested.

The next node is immunometabolism. Upon protective activation, the macrophage switches to aerobic glycolysis (reminiscent of the Warburg effect of tumor cells), with accumulation of succinate, stabilization of HIF-1α, and production of IL-1β; in this state, the cell restricts the growth of Mtb more effectively [49]. These data were obtained mainly in human alveolar macrophages and in experimental models, so their extrapolation to other populations and stages requires caution. An alternative scenario is also possible—active oxidative metabolism and the accumulation of lipids (triglycerides and cholesterol esters), with the formation of a foamy macrophage characteristic of granulomatous inflammation, in which an environment favorable to the long-term persistence of Mtb is created [48,50,51].

The functional polarization of the macrophage is closely tied to switching among the glycolytic, mitochondrial, and lipid-dependent operating modes of the cell. The traditional division into M1 and M2 is convenient as a simplified, historical model (M1-like IFN-γ activation is more bactericidal, whereas M2-like states control Mtb less well), but it is too coarse to describe granulomatous inflammation: single-cell sequencing data show that pro-inflammatory, hypoxic, profibrotic, mixed, and foamy macrophage states coexist within a single lesion, shaped by local oxygen, cytokines, lipids, and bacterial burden [2,52,53,54]. Equally important is the manner in which infected cells die: apoptosis is usually regarded as the more favorable option (the bacterium remains within apoptotic bodies, and the material is used for cross-presentation), whereas necrosis, necroptosis (RIPK1 → RIPK3 → MLKL), pyroptosis (caspase-1/GSDMD), and ferroptosis release the pathogen and danger signals, amplifying inflammation and spread. Mtb appears able to shift macrophage death away from apoptosis toward the more destructive options by exploiting ESX-1 and the lipid components of its cell wall [2,127,128,129].

Finally, the host cell itself changes over time: in an experimental model, during the first day Mtb resides predominantly in alveolar macrophages, where its growth is restricted, and after several days it moves into the interstitium—into interstitial and recruited monocyte-derived macrophages, where it replicates more actively and more readily reaches the lymph nodes; this is consistent with the differences in the transcriptional programs of alveolar and interstitial macrophages (described in a murine model) [2,16,17,18].

Relevance to the COPD–TB link. The nodes listed above matter not in themselves but because they are precisely the ones disrupted by smoking and COPD. In COPD, the macrophage-level events—less effective phagocytosis, an efferocytosis defect, a metabolic shift, and impaired resolution of inflammation—are not isolated deviations but components of a more general failure of intracellular control, clearance of dead cells from the tissue, termination of inflammation, and repair. At the same time, early signals from γδ T cells, above all IFN-γ and TNF, can support the protective activation of the macrophage [103].

## 4. Mycobacteria and the Airway and Alveolar Epithelium

For a long time, the lung epithelium was perceived merely as a mechanical barrier. That view has changed: epithelial cells are now regarded as an active regulator of the early outcome of Mtb infection. They recognize mycobacteria, secrete antimicrobial molecules, generate a chemokine signal, and initiate early inflammation [56]. The central thesis is that the epithelium determines what fraction of the pathogen reaches the phagocytes at all, and in what form—and that in COPD it is precisely this regulatory layer that fails (“barrier failure”). At the same time, the epithelium does not work solely in the host’s favor: under certain conditions, Mtb uses epithelial cells for entry, intracellular persistence, and possibly early translocation [4,24].

The cellular composition of the epithelium changes along the airways: in the conducting regions, basal, ciliated, goblet, and club cells predominate, whereas in the alveoli there are type I and type II alveolar epithelial cells (AT1 and AT2 cells) [21]. Of particular importance are AT2 cells: they synthesize surfactant, maintain alveolar homeostasis, and serve as precursors of AT1 cells. For Mtb, these cells matter not only as an element of the barrier—the bacterium has been shown to be able to enter AT2 cells and replicate within them [24], and in the A549 alveolar epithelial model, intracellular growth is accompanied by a change in the bacterium’s transcriptional profile [57]. The alveolar epithelium therefore probably participates in early translocation events, although the main route of spread is still attributed to infected phagocytes [4].

In the alveolar environment, the surfactant collectins SP-A and SP-D are of considerable importance. Their action on Mtb is bidirectional: SP-A enhances uptake of the bacterium by human macrophages [58], including through increased activity of the mannose receptor [59], but enhanced phagocytosis is not equivalent to killing and may facilitate entry of the pathogen into the cell; SP-D, by contrast, reduces uptake of Mtb [60] and promotes fusion of the phagosome with the lysosome [61]. Collectins are therefore more accurately viewed as regulators of the early contact between Mtb and phagocytes rather than as simple antimicrobial proteins [23], although their contribution is not decisive—in mice, the absence of SP-A and SP-D did not impair control of aerosol infection [62].

The epithelium also possesses its own antimicrobial activity: it produces short cationic peptides that damage bacterial membranes [19]. In response to Mtb, the alveolar epithelium expresses β-defensin-2 [63]. Activation of TLRs triggers a vitamin D-dependent antimicrobial program [64], and antimicrobial activity against Mtb requires induction of the cathelicidin LL-37 [65]. Alongside this, the epithelium responds rapidly to mycobacteria by producing cytokines and chemokines, attracting neutrophils, monocytes, and lymphocytes and setting the initial character of inflammation [66]. This cytokine response apparently also participates in the early activation of γδ T cells at barrier surfaces, which makes the “epithelium–γδ T cell” interaction one of the likely early elements of the local response.

In the conducting airways, defense is complemented by club and basal cells and by mucociliary clearance. Club cells secrete CC16 (SCGB1A1), an anti-inflammatory protein whose level reflects the state of the epithelium and declines upon injury [67,68]; basal cells provide for regeneration of the barrier [20]. Mucociliary clearance removes some of the mycobacteria even before contact with cells: mucus binds the microorganisms, and the ciliated epithelium clears them out [69]; the gel is based on the mucins MUC5B/MUC5AC, and MUC5B is required for normal clearance of the airways [70,71]. With prolonged exposure to tobacco smoke, this system shifts in an unfavorable direction: the reparative program is disrupted and may progress to squamous or mucous metaplasia, clearance deteriorates, and the pathogen is more likely to be retained in the airways.

Certain epithelial mechanisms are exploited by Mtb directly to its own advantage. M cells normally transport antigens across the epithelium, but the mycobacterium is able to use this route for entry and the initiation of infection [72]. External factors alter the properties of the epithelium: in a cell model, nicotine accelerates the intracellular growth of Mtb, especially in AT2 cells, and at the same time reduces the production of β-defensins and LL-37 [73]. Tobacco smoke thereby weakens precisely those mechanisms of early epithelial defense that ought to restrict Mtb before macrophages and T cells are engaged.

Taken together, the epithelium participates in anti-TB defense in several ways—it maintains mucociliary clearance, produces surfactant proteins, antimicrobial peptides, cytokines, and chemokines, and restores the barrier after injury—but in COPD this system fails: clearance deteriorates, antimicrobial activity declines, repair is impaired, and metaplasia increases. The epithelium probably also participates in early interaction with γδ T cells; however, direct data on the “epithelium–γδ T cell–Mtb” dialogue, specifically in combination with COPD, remain limited, and this should be designated as a gap rather than an established mechanism.

## 5. The αβ T Cell Response to Mtb

If the macrophage provides the initial stage of intracellular interaction with Mtb, durable control of the infection depends on a coordinated αβ T cell response: it activates macrophages, sustains the granuloma, and restricts dissemination. This response has three features that are fundamental in TB—it develops with a delay, depends on the localization of the cells within the tissue, and requires fine regulation, because excessive inflammation itself damages the lung. Only the classical αβ T cells are considered below.

The central element of acquired defense is the CD4^+^ T cell, acting mainly through the IL-12 → IFN-γ → macrophage activation axis [2]. Its importance has been confirmed at various levels of evidence: in mice, the absence of IFN-γ leads to rapid dissemination of the pathogen [75,76]; in humans, inborn defects of the IL-12/IFN-γ pathway (Mendelian susceptibility to mycobacterial disease, MSMD) cause severe infections even from weakly virulent mycobacteria and from BCG [77,78]; IFN-γ from CD4^+^ T cells is required for host survival and for support of CD8^+^ cells [79]; and in human immunodeficiency virus (HIV) infection, the selective depletion of Mtb-specific CD4^+^ T cells sharply increases the risk of TB [80,81]. This axis alone, however, is not sufficient. One of the key features of TB is the late engagement of acquired immunity: specific T cells appear in the lung only after 2–3 weeks, because naive cells are activated not in the tissue but in the draining lymph node, to which the antigen is delivered by a dendritic cell [82]; during this time, Mtb becomes established in early cellular reservoirs and begins to disseminate [83]. It is for precisely this reason that early reactions, including the γδ T cell response and epithelial–macrophage signals, take on particular importance even before αβ T cell control has formed.

The CD4^+^ response is not limited to the Th1 program. The IL-23 → IL-17 (Th17) pathway also participates at the early stage of inflammation and in cell recruitment, and after vaccination it promotes a more rapid influx of IFN-γ^+^ CD4^+^ T cells into the lung [84]; at the same time, an excess of IL-17 serves as a bridge to neutrophilic inflammation and tissue damage. Regulatory T cells, by contrast, restrain the response and may delay the early effector stage (in a murine model, Foxp3^+^ Tregs accumulate in the lungs and draining lymph nodes) [85]. CD8^+^ T cells complement the defense: they kill infected cells using perforin and granzymes, produce IFN-γ, and, in humans and primates, employ granulysin, which damages the mycobacterium itself [86]; their depletion worsens control of infection in primates [87], and their function depends in part on CD4^+^ cells and IFN-γ [79,88]. Importantly, granulysin is characteristic of humans and primates and is absent in ordinary laboratory mice, and in an already-damaged lung a chronic cytotoxic response may injure the tissue without a guaranteed improvement in control of Mtb.

In pulmonary TB, what is decisive is not only the magnitude of the response but also its spatial organization. Some of the cells remain in lung tissue as CD4^+^ tissue-resident memory T cells (TRM) [27]; in latent TB infection, a local accumulation of such TRM cells in the airways has been described [26]. It has been established experimentally that CD4^+^ T cells migrating into the parenchyma (CXCR3^+^KLRG1^−^) control Mtb better than those remaining in the vasculature (KLRG1^+^) [89]; the protective role of TRM cells is well studied in respiratory viral infections, but these data should be extrapolated to TB with caution [25]. The practical conclusion is important for vaccines: a promising vaccine should generate not only circulating T cells but also local T cells of the airways and parenchyma.

Finally, the αβ T cell response in TB must be not merely strong but balanced: insufficient activation fails to control Mtb, whereas an excessive or improperly localized T cell reaction intensifies inflammatory damage to lung tissue. Thus, terminally differentiated KLRG1^+^ T cells usually possess a lower protective potential [89], whereas less differentiated PD-1^+^ T cells may be more effective in controlling infection; in TB, PD-1 is not only a marker of exhaustion but also a regulator that limits damaging inflammation—its absence in mice worsens the course of disease because of an uncontrolled CD4^+^ response [90]. The same principle is seen in the case of TNF: it is required for defense and for maintenance of the granulomatous response [91], and its blockade during anti-TNF therapy can reactivate latent TB [92]; overall, in mycobacterial infection there is an optimum of inflammation, and deviation in either direction worsens the outcome [93]. Moreover, even functionally active T cells do not always reach the pathogen: they are more often located at the periphery of the lesion, and granulomas are heterogeneous—within a single host, some lesions are sterilized while others progress [52,53,54,127].

Consequently, αβ T cells are necessary for the long-term control of Mtb, but their response is delayed and depends on localization; protection requires not the maximal intensity of inflammation but a properly organized reaction. The delay of the αβ response leaves an early time window in which rapid cellular populations, including γδ T cells, are especially important. In COPD, this balance shifts: chronic cytotoxicity and inflammation intensify, whereas the protective organization of the response may be disrupted.

## 6. γδ T Lymphocytes: An Early Link Between the Epithelial Barrier, the Macrophage, and Adaptive Immunity

γδ T lymphocytes are of interest in TB because they occupy an intermediate position between the rapid innate recognition of antigens and the classical adaptive response. Antigen-specific αβ T cells are necessary for the long-term control of Mtb, but they arrive at the lesion with a delay, when the pathogen has already become established in macrophages and other cells. Phosphoantigen-reactive Vγ9Vδ2 cells are positioned to respond during this interval: they react to microbial metabolites of the isoprenoid pathway and to signals of cellular stress faster than the classical response forms [82,98,100,103]. Whether they functionally compensate for the delayed αβ T cell response in human TB remains untested. This system is characteristic of humans and non-human primates and has no direct counterpart in the mouse. γδ T cells should therefore be regarded not as a universal, principal mechanism of defense but as an early link connecting macrophage infection, epithelial stress, the response to BCG, and the inflammatory changes of COPD.

### 6.1. Subsets of γδ T Cells

Unlike αβ T cells, γδ T cells carry a T cell receptor (TCR) composed of γ and δ chains, which allows them to combine clonal recognition of antigen with rapid tissue surveillance and a reaction to signals of cellular stress [96,97]. In TB, it is useful to distinguish several functionally and evolutionarily distinct groups of γδ T cells. The first is the Vγ9Vδ2 cells, which predominate among the circulating γδ T cells of human blood and have a functional counterpart in non-human primates; they react to pAgs of microbial and endogenous isoprenoid metabolism. The second is the tissue non-Vδ2 γδ T cells, predominantly Vδ1 cells, which are more often associated with epithelial and barrier niches and can recognize stress-induced molecules, lipid antigens, and other non-classical ligands, including in the context of CD1. The third is the murine IL-17-producing γδ subpopulations, which are useful for studying the effector role of IL-17 but do not reproduce the human Vγ9Vδ2 system. In lung tissue, the composition of γδ T cells is variable and depends on the anatomical zone, age, inflammatory context, and method of analysis [31].

### 6.2. Well-Established Evidence: Phosphoantigen Recognition

The recognition mechanism is the most reliably established. Vγ9Vδ2 cells respond to pAgs—small phosphorylated intermediates of isoprenoid synthesis: the microbial non-mevalonate (MEP) pathway yields the potent HMBPP, whereas the endogenous mevalonate pathway produces the weaker IPP [98,99]. Phosphoantigens are not presented by human leukocyte antigen (HLA) molecules; their recognition is linked to butyrophilins: BTN3A1 binds the pAg via its intracellular B30.2 domain and signals metabolic stress [28,100,101], while BTN2A1 interacts directly with the germline-encoded regions of the Vγ9Vδ2 TCR and is required, together with BTN3A1, for activation [29,32,102]. Through this axis, the Vγ9Vδ2 cell receives a signal that microbial or stress-induced metabolites have accumulated inside another cell, without classical HLA-dependent presentation.

### 6.3. Reasonably Strong Evidence: Rapid Effector Functions

After activation, γδ T cells rapidly engage their effector mechanisms. In mycobacterial infection, Vγ9Vδ2 cells proliferate and acquire a multifunctional phenotype, which has been shown in humans and primates [30,103]. Their early production of IFN-γ and TNF can enhance the antimicrobial activation of macrophages before the arrival of a full αβ T cell response. γδ T cells also possess direct anti-mycobacterial activity, and here the distinction between extracellular and intracellular bacteria matters. Granulysin is a cationic protein of cytotoxic granules that inserts into and disorganizes the mycobacterial envelope: purified granulysin killed extracellular Mtb by altering the membrane integrity of the bacillus, whereas reducing the viability of intracellular Mtb required granulysin together with perforin, the latter presumably giving the effector molecule access to the bacterium within the infected cell [86]. Vγ9Vδ2 cells reproduce this activity against both intracellular and extracellular Mtb [106], and their functional repertoire, under certain conditions, includes features of antigen presentation [97,103]. Recognition through the T cell receptor is not the only input: Vγ9Vδ2 cells also carry activating natural killer receptors, chiefly NKG2D and DNAM-1, which set the threshold at which pAg sensing is converted into effector function [97,138]; direct engagement of NKG2D upregulates CD69 and CD25 and triggers TNF production and the release of cytolytic granules, although not IFN-γ secretion [139]. Because the ligands of these receptors are stress-induced, this second layer of activation would be expected to be engaged in chronically injured lung tissue, but it has not been examined there.

The evidence for this mechanism is drawn predominantly from human in vitro systems and from primate studies. Granulysin has no functional counterpart in ordinary laboratory mice [140], so the murine literature cannot be used to weigh its contribution, and the mechanism has not been examined in patients with coexisting COPD and TB.

IL-17 requires separate and more cautious treatment. It is a context-dependent mechanism rather than unambiguously protective or damaging. In a murine model, early IL-17 production is determined predominantly by γδ T cells rather than by CD4^+^ cells [104]; in humans, IL-17^+^ γδ T cells are elevated in active pulmonary TB [105]. At an early stage, IL-17 helps to organize the response and attract cells to the lesion, but its excess serves as a bridge to neutrophilic inflammation and tissue damage. The IL-17-associated function of γδ T cells should therefore be interpreted according to stage and tissue context—as a mechanism that can both protect and sustain immunopathology. IL-22, which is frequently co-produced with IL-17 at barrier surfaces, requires a more cautious statement still. IL-22 and its receptors are raised in the lungs of patients with COPD and in cigarette-smoke-induced experimental disease, in which γδ T cells are one of several cellular sources and IL-22 deficiency protects against emphysema-like alveolar enlargement [141]; γδ T cells also secrete IL-17A and IL-22 in explanted human emphysematous lung [142]. The IL-17^+^ Vγ9Vδ2 subset differentiated in vitro, however, produces IL-17 without IL-22 [143], so IL-22 in the lung is more likely to derive from tissue γδ populations than from the circulating pAg-reactive subset, and it has not been measured in TB–COPD.

### 6.4. Evidence from Primate Models: Protective Potential

The most direct evidence of a protective role has been obtained in non-human primates. Activation and differentiation of Vγ2Vδ2 cells by a pAg in combination with IL-2 increased resistance to TB [107]; immunization that generates a sustained Vγ2Vδ2 effector response improved control of infection [5]; and adoptive transfer of pAg-specific γδ T cells attenuated Mtb infection [108]. These data are valuable because they pertain to a system close to the human Vγ9Vδ2 axis, but they should be interpreted specifically as data from primate models rather than as direct clinical proof.

### 6.5. Moderately Strong Evidence: A Role in the Response to BCG

BCG vaccination also engages γδ T cells. In humans, it enhances their reactivity to mycobacterial antigens, and the response shows features of memory [109]; the combination of a rapid response and memory-like traits makes γδ T cells a promising target of vaccine strategies [110]. More recent data extend the picture beyond Vγ9Vδ2: BCG can induce antibacterial functions in tissue Vδ1/Vδ3 cells that are associated with protection [112]. This result should be interpreted with caution—the work is recent, and the conclusions are largely associative.

### 6.6. Limitations of Murine Models for Studying the Vγ9Vδ2 Response

The extrapolation of conclusions about γδ T cells from murine models to humans requires particular caution, and this issue deserves separate attention. The system central to humans and primates—the pAg-reactive Vγ9Vδ2 cells that recognize HMBPP/IPP through the BTN3A1/BTN2A1 axis—is absent in ordinary laboratory mice: the mouse has no functional equivalent of the Vγ9Vδ2 TCR or of the corresponding butyrophilin recognition system [100,101,102,138,144,145,146]. Murine γδ T cells are informative primarily for studying IL-17-dependent effector programs and tissue surveillance, but they do not model pAg recognition or the protective potential associated with it. A similar limitation applies to granulysin, which mediates part of the direct anti-mycobacterial activity in humans and primates and is absent in ordinary mice [106,140]. The key mechanistic claims about Vγ9Vδ2 therefore rest on studies in humans, primates, and in vitro systems, whereas murine data are used as supplementary and are interpreted with interspecies differences in mind [138,144,145,146].

Set against one another, the three systems answer different questions. Mice remain the only model in which gene deletion, adoptive transfer and controlled aerosol challenge can be combined at scale, and they established the non-redundant roles of IFN-γ and TNF [75,76,91] and the dominance of γδ T cells as an early source of IL-17 [104]; what they cannot reproduce is pAg sensing [100,101,102,145,146], granulysin-dependent killing [106,140], or the caseating, hypoxic granuloma of human disease [10,53]. Non-human primates reproduce the Vγ2Vδ2 axis, the full spectrum of granuloma types and the latency-to-reactivation continuum [30,53], and it was in macaques that targeted expansion of Vγ2Vδ2 cells [107,108] and route-dependent BCG protection [147,148] were demonstrated; cost and ethical constraints, however, keep sample sizes small and make replication difficult. Human studies carry the greatest translational weight but are largely observational, are dominated by peripheral blood rather than lung tissue [103,105], and rarely include the comorbid populations of interest [149,150]. No single system is adequate on its own, which is why the mechanistic claims made in this review are qualified throughout by the system in which they were obtained.

### 6.7. Localization, Limited COPD Data, and Perspectives

In the lung, γδ T cells are located predominantly at barrier surfaces—next to the epithelium and within the mucosa—which allows them to respond early to microbial molecules and to stress signals from infected or dying cells [31,97]. Epithelial cytokines probably participate in their activation, reflecting the “epithelium–γδ T cell” interaction.

The role of γδ T cells in COPD has been insufficiently studied. On the one hand, in patients with COPD a reduction in the number of T cells with innate-response features has been described [39]; that study enumerated invariant natural killer T (iNKT) and mucosal-associated invariant T (MAIT) cells rather than γδ cells, but the studies that measured γδ T cells directly point in the same direction. The expansion of γδ T cells seen in the blood and bronchoalveolar lavage of smokers with preserved lung function is blunted in patients with COPD [149], and γδ T cell numbers are reduced in induced sputum and bronchoalveolar lavage in COPD relative to asthma and to healthy subjects, correlating inversely with FEV1 and with pack-years of smoking [150]. Taken together, these changes may weaken early barrier defense. On the other hand, γδ T cells are able to sustain pathological inflammation: in a model of emphysema, through IL-17A they stimulated epithelial and stromal cells to produce granulocyte colony-stimulating factor (G-CSF), promoting the generation of pathogenic neutrophils [111]. These observations do not prove a direct mechanism in the combination of COPD and Mtb infection, but they do suggest that in COPD the γδ T cell response may shift between deficient early defense and IL-17-dependent neutrophilic inflammation. Therapeutic targeting of γδ T cells specifically in the combination of COPD and TB remains hypothetical at present.

In addition, murine models are poorly suited to studying the Vγ9Vδ2 system; the role of tissue non-Vδ2 γδ T cells in Mtb infection is less well studied than that of circulating Vγ9Vδ2 cells; and it is also unclear how COPD alters the γδ response specifically in TB. In this regard, promising directions include vaccines that generate an early local γδ response, immunotherapy involving the expansion of Vγ9Vδ2 cells with pAgs or aminobisphosphonates, and the use of Vγ9Vδ2 dynamics as a possible biomarker [103,110].

**Figure 2:**
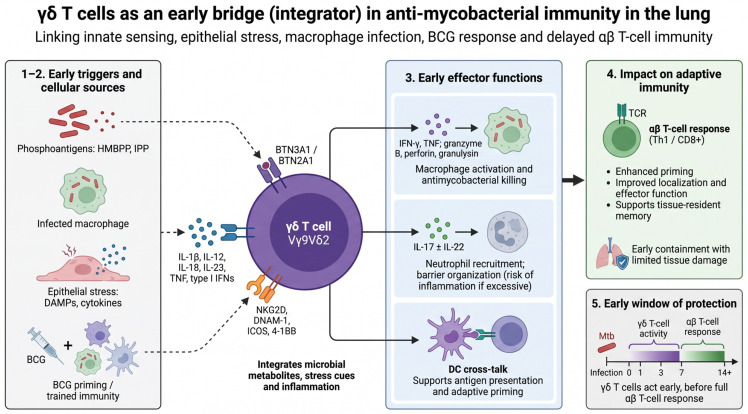
Proposed integrative role of γδ T cells in the early anti-TB response, linking innate sensing, epithelial stress, macrophage infection, the response to BCG, and delayed αβ T cell immunity. The scheme is a working model: pAg recognition and the effector functions shown are supported by direct evidence, whereas the integrative role itself is inferred. Arrows indicate the direction of the proposed interactions: dashed arrows represent upstream activating inputs to γδ T cells, whereas solid arrows represent downstream effector and immunoregulatory effects. Created in BioRender. Oskin, D. (2026) https://BioRender.com/uk18e1f (accessed on 31 July 2026).

Taken together, without being a universal, principal mechanism of defense, γδ T cells occupy an important place in the early response. The Vγ9Vδ2 cells of humans and primates recognize microbial metabolites through the BTN3A1/BTN2A1 axis (well established) [28,29,32,100,101,102], rapidly produce IFN-γ, TNF, and IL-17 and exhibit granulysin-dependent activity (reasonably well established) [30,104,105,106], participate in the response to BCG with features of memory (moderately well established), and improve control of infection in primate models [5,107,108]. In COPD, their role is probably dual (limited data), and their therapeutic application in COPD–TB is hypothetical. Direct data for the COPD–γδ T cell–Mtb axis are as yet lacking. These relationships, and the boundary between established mechanisms and inference, are summarized in Figure 2; the evidence underlying each claim is graded in Table 2.

## 7. BCG as a Tool for Studying Cellular Immunity

New data underscore the interest in BCG not only as a subject of vaccinology but also as a tool that demonstrates how incompletely the classical scheme of Th1 immunity explains anti-TB defense. This live attenuated strain of *Mycobacterium bovis* reliably reduces the risk of severe forms of TB in children, whereas protection against pulmonary TB in adults varies substantially between populations [151]; BCG may also partially reduce the risk of Mtb infection, although the robustness and reproducibility of this effect are debated [152]. This alone indicates that BCG engages not only the antigen-specific Th1 response but also innate, T cell, and tissue mechanisms.

The first important conclusion from BCG studies relates not so much to the magnitude of the IFN-γ response as to the quality and localization of the immune reaction. The level of IFN-γ production in itself poorly reflects the degree of protection; what proves more meaningful is the presence of effector cells in the regions of the lung where the infectious process begins. This has been clearly shown in experiments in macaques: intravenous administration of BCG provided more pronounced protection than standard intradermal vaccination [147], and the protective effect was associated with the accumulation of antigen-specific T cells and NK cells in the airways, whereas peripheral-blood parameters were less informative [148]. Direct transfer of an intravenous vaccination regimen to clinical practice is not feasible; the principle itself, however, is important: in TB, what matters is not only the fact that a T cell response forms but also its localization in the regions of the lung where early contact with Mtb occurs.

The second and most substantial conclusion concerns the concept of trained immunity: after a primary stimulus, cells of the innate compartment respond more strongly to subsequent stimuli, including unrelated challenges, owing to stable epigenetic and metabolic changes in the cells rather than to the classical memory of T and B cells. In human monocytes, BCG induces NOD2-dependent reprogramming and enhances cytokine production upon restimulation [153], which has been described as a program of innate memory linked to a shift toward glycolysis [45]; a similar “trained” reactivity has been found in NK cells [154]. The effect is not limited to mature blood cells: in an experimental model, BCG reprograms hematopoietic stem cells, which give rise to macrophage progeny with enhanced antimicrobial activity [155], and in humans a reprogramming of hematopoietic progenitors after vaccination has been described [156]. Clinical relevance is supported by a study using controlled infection with the yellow fever vaccine virus: in BCG-vaccinated subjects, viremia was lower, which coincided with cytokine signatures of trained immunity [157]. After BCG vaccination, human γδ T cells show an enhanced response to mycobacteria and acquire features of a memory-like reaction [109], and more recent data extend this beyond Vγ9Vδ2—to tissue Vδ1/Vδ3 cells with antibacterial functions associated with protection [112]. Taken together, this supports the view of BCG as a stimulus that engages not only the classical αβ T cells but also rapid T cell populations, including γδ T cells [110].

With regard to COPD, the data on BCG and BCG-induced trained immunity should be interpreted with caution. At present, there is no direct evidence that BCG reduces the risk of developing COPD or alters the course of already-established disease. The possible association remains indirect and may be bidirectional in character. On the one hand, in individual studies BCG has been associated with a reduced frequency of respiratory infections, which are common triggers of COPD exacerbations; however, the effects of vaccination are inconsistent and depend on age, baseline immune status, the vaccine strain, and the regimen of administration [158,159,160]. On the other hand, trained immunity engages myeloid cells, NK cells, and γδ T cells—populations whose functional state is often altered in COPD. Therefore, an enhancement of early innate reactivity in already-damaged lung tissue might, in theory, not only improve control of infections but also sustain neutrophilic inflammation. Consequently, the use of BCG for the prevention or modification of COPD should be regarded not as a proven method but as a hypothesis requiring separate clinical validation. A more general conclusion from studies of BCG and of new anti-TB vaccines is that the assessment of their efficacy should not be confined to the IFN-γ response in peripheral blood. Thus, contemporary strategies—including BCG revaccination [161], the subunit vaccine M72/AS01E, which has shown approximately 50% protection against progression from latent infection to active TB [162], and other vaccine platforms [163]—are increasingly being considered in light of their ability to generate local airway immunity, trained myeloid reactivity, and a response of innate-like lymphocytes, including NK and γδ T cells.

Two aspects of this literature warrant a more critical reading than they usually receive. The first is mechanism. Training of monocytes rests on chromatin remodeling at the promoters and enhancers of inflammatory genes—enrichment of H3K4me3 and H3K27ac and increased chromatin accessibility—coupled to a metabolic shift toward glycolysis and glutaminolysis and initiated through NOD2 sensing of mycobacterial muramyl dipeptide [153,164]; when equivalent marks are laid down in bone marrow progenitors, the trained phenotype outlives the short lifespan of circulating monocytes [155,156]. The second is durability and clinical benefit. Enhanced ex vivo cytokine responses have been documented over periods of months rather than years, they attenuate with time and with age, and their relation to clinical protection is inconsistent: BCG lowered viremia in a controlled yellow fever vaccine challenge [157] but produced heterogeneous results against respiratory illness in adults [160]. Most directly, a phase 2b trial in interferon-γ release assay (IGRA)-negative adolescents found no protection from BCG revaccination against sustained IGRA conversion (vaccine efficacy −3.8%, 95% CI −48.3 to 27.4), with more frequent injection site reactions in the BCG arm [165]. An earlier phase 2b trial had reported 45% efficacy (95% CI 6 to 68) against the same end point [161]; the confirmatory trial did not reproduce this [165], and the discrepancy itself illustrates how fragile inference from immunogenicity to protection remains. BCG revaccination should therefore be described as investigational and population-dependent rather than promising. Because BCG is a live vaccine it is also contraindicated in significant immunosuppression, and the theoretical concern that heightened myeloid reactivity might aggravate neutrophilic inflammation is of particular relevance in COPD, where that axis is already engaged. The evidence on BCG-induced cellular immunity, and its limits, is summarized in Table 3.

## 8. Mycobacterial Antigens as a Tool for Studying Cellular Immunity

Mycobacterial antigens are important not only as diagnostic and vaccine targets but also as factors that determine the character of the cellular immune response. Protein antigens predominantly engage MHC-dependent αβ T cells, lipids and lipoglycans engage CD1-restricted and iNKT cells, and pAgs engage Vγ9Vδ2 γδ T cells, whereas whole-cell preparations activate a broader, multicellular response, including the mechanisms of trained immunity.

### 8.1. Protein Antigens → the αβ T Cell Response

Protein antigens are recognized in the context of major histocompatibility complex (MHC) and activate the classical αβ T cells. Historically, the first reagent was tuberculin purified protein derivative (PPD): the delayed-type skin reaction revealed the fact of an encounter with mycobacteria but, owing to its complex and poorly standardized composition, had low specificity (the reaction is also possible after BCG vaccination or contact with non-tuberculous mycobacteria). A fundamental shift came from comparative genome analysis: the proteins ESAT-6 and CFP-10 are encoded by the RD1 region, are linked to the ESX-1 system, and are absent from BCG and from most non-tuberculous mycobacteria [114], which made them more specific markers on which the IGRA tests are based [115]. However, IGRAs detect sensitization but do not reliably distinguish latent infection from active disease and do not indicate whether the response is protective. The Ag85 family (A/B/C)-secreted mycolyltransferases with marked immunogenicity [113] served as the basis for the MVA85A vaccine, which proved safe and immunogenic but provided no additional protection in children previously vaccinated with BCG [117]. This result became an important practical lesson for the entire field: a strong Th1 response does not equal protection [116]. Against this backdrop, the subunit vaccine M72/AS01E afforded moderate protection against progression from latent infection to active TB [162]. Consequently, the significance of a protein antigen is determined not only by its immunodominance but also by the mode of delivery, the adjuvant, the tissue localization of the response, and the baseline state of the immune system.

### 8.2. Lipids and Lipoglycans in CD1-Restricted and iNKT-Cell Responses

Non-protein antigens expanded the understanding of immunity beyond the “peptide–MHC” scheme. Mycolic acids can be recognized by T cells in the context of CD1-group molecules, which demonstrates the existence of a lipid-specific T cell response in TB [36]. Mannose-capped lipoarabinomannan (ManLAM) acts not so much as a conventional antigenic target as a factor of immune modulation: through DC-SIGN, mycobacteria suppress the function of dendritic cells, and LAM and related lipoglycans participate in the disruption of phagosome maturation [118,119]. At the same time, LAM has proven useful for diagnosis: it is excreted in the urine and is used in non-invasive tests, especially in HIV-associated TB [166]. Lipid antigens thereby characterize both a distinct (CD1-restricted) level of recognition and mechanisms of immune modulation.

### 8.3. Metabolites and Phosphoantigens → Vγ9Vδ2 γδ T Cells

Phosphoantigens—small non-peptide phosphorylated metabolites of isoprenoid metabolism—are of particular importance. Isopentenyl pyrophosphate (IPP) and related prenyl pyrophosphates were identified as natural mycobacterial antigens recognized by human Vγ9Vδ2 γδ T cells; it was precisely this class of ligands that made it possible to define the Vγ9Vδ2 population functionally, through its ability to react to non-peptide pAgs [99]. It was later established that the strongest stimuli are associated with the microbial non-mevalonate (MEP) pathway of isoprenoid biosynthesis [98], which links the Vγ9Vδ2 response not merely to the presence of a microbial antigen but to a metabolic feature of the bacterium. From an experimental standpoint, this is a key tool: pAgs, their synthetic analogues, and aminobisphosphonates make it possible to activate and differentiate Vγ9Vδ2 cells in a targeted manner and to study their potential, connecting the antigenic characterization of Mtb with the central theme of this review—the role of γδ T cells.

### 8.4. Whole-Cell Preparations → Trained and Multicellular Responses

A separate group consists of whole-cell mycobacterial preparations, above all inactivated *Mycobacterium vaccae*. Unlike individual antigens, such preparations act not through a single defined specificity but through the combined effect of numerous mycobacterial components on the innate and adaptive arms of the immune response. It is hypothesized that this may favor a shift in the immune reaction toward a Th1-oriented profile. However, the clinical evidence base remains ambiguous: a Cochrane review did not confirm convincing benefit for the principal clinical outcomes [167], whereas a later meta-analysis pointed to a possible improvement in intermediate measures, including sputum conversion and radiographic dynamics [168]. *M. vaccae* should therefore not be regarded as a proven therapeutic approach, but it remains an instructive example of how a whole-cell mycobacterial stimulus can alter the overall profile of the immune response, and it converges methodologically with the concept of trained immunity.

Thus, mycobacterial antigens can be regarded as tools that allow the different levels of the cellular response to Mtb to be assessed separately. PPD reflects the fact of prior sensitization; ESAT-6 and CFP-10 are used to detect a more specific αβ T cell response in IGRA tests; the experience with Ag85 and MVA85A shows that marked immunogenicity does not necessarily mean effective protection; and M72/AS01E demonstrates the potential of rationally designed antigen combinations. Lipid antigens make it possible to analyze the CD1-restricted T cell response, and pAgs make it possible to study Vγ9Vδ2 cells directly. At the same time, the influence of COPD, smoking, and post-TB remodeling of lung tissue on antigen- and pAg-specific reactions remains insufficiently studied. This gap has practical significance both for the interpretation of IGRAs in patients with chronic lung disease and for the development of vaccine strategies oriented toward local and early cellular immunity.

## 9. COPD-Associated Molecular Remodeling of Anti-Mycobacterial Immunity

It was shown above that anti-TB immunity cannot be considered separately from the state of the lung tissue itself: the work of macrophages, the epithelium, αβ and γδ T cells, and the effect of BCG all depend on the local environment. COPD represents one such altered environment. In molecular terms, COPD is associated with alterations in the same host–Mtb interfaces outlined above—epithelial antimicrobial peptides, macrophage immunometabolism and efferocytosis, the pool of rapid innate-like T cells, and the IL-17/neutrophil axis. In COPD, anti-mycobacterial immunity shifts simultaneously in two directions—the early protective mechanisms weaken, while the damaging ones intensify. This is because COPD is characterized by profound tissue remodeling: persistent inflammation, most often associated with tobacco smoke, is accompanied by changes in the small airways, damage to the epithelium, accumulation of macrophages and neutrophils, an increased proportion of cytotoxic T cells, and emphysema [55,122]. Mtb may therefore encounter not intact tissue but an environment in which part of the early defense is weakened, and the damaging reactions are already activated.

### 9.1. Clinical and Epidemiological Evidence for the TB–COPD Relationship

Epidemiological data support these links. Smoking is consistently associated with an approximately twofold increase in the risk of TB [6,7], and a population-based cohort study showed that COPD itself is independently associated with an increased risk of active TB [8]. COPD may therefore be regarded as a state in which the probability of Mtb becoming established in lung tissue is increased.

Three clinical situations are often merged in this literature and are better kept apart. In the first, COPD precedes active TB: population-based cohorts show an independent excess risk of roughly twofold that persists after adjustment for smoking [8,12], although age, socioeconomic position and exposure to inhaled and systemic corticosteroids remain incompletely controlled confounders, and few of these studies used post-bronchodilator spirometry to define COPD. In the second, TB precedes obstruction: a systematic review of lung function after treated pulmonary TB found persistent ventilatory defects in a substantial proportion of microbiologically cured patients, with obstructive, restrictive and mixed patterns all represented [13], and cohort data link TB acquired during the developmental period to COPD and pre-COPD in mid-to-late adulthood [9]. In the third—patients in whom the two diseases coexist—the evidence is thinnest. Pooled estimates indicate that COPD is present in about 16% of patients with TB and that TB has occurred in about 6% of patients with COPD [12], so the overlap is common; yet the constituent studies are heterogeneous in how COPD was ascertained and almost none report immune phenotyping. Transcriptomic comparison of the two conditions has begun to identify shared molecular programs [169], but we are not aware of a clinical study that has characterized Vγ9Vδ2 or tissue non-Vδ2 cells specifically in patients with both COPD and TB. This is the central evidence gap addressed by the present review: it is a gap in the primary literature rather than an omission in the argument developed here, and it defines what the model proposed below is and is not entitled to claim.

### 9.2. Heterogeneity of COPD and Its Consequences for the Evidence Base

COPD is not a single disease, and the immunological observations summarized here were not all made in the same kind of patient. Current nomenclature separates COPD by cause—genetic, developmental, cigarette-smoke-related, biomass- and pollution-related, infection-related (including post-TB), asthma-associated, and of unknown cause—precisely because these routes differ in their pathology [170]. The distinction matters here in three ways. First, most of the mechanistic work cited in this review—impaired phagocytosis and efferocytosis, CD8^+^ accumulation, elastase- and MMP-12-driven matrix destruction—derives from tobacco-related, emphysema-predominant disease and from cigarette smoke or elastase models and cannot be extended to the syndrome as a whole. Second, biomass-related COPD, which predominates in many high-TB-burden settings, shows a different balance: more small-airway and bronchiolar remodeling and mucus hypersecretion, relatively less emphysema, and a distinct oxidative and inflammatory profile [171]. Third, post-TB COPD is itself structurally distinct, with fibrosis, bronchiectasis and cavitary destruction contributing alongside airflow limitation [11,13], so that treating it as a proxy for smoking-related COPD confuses cause with consequence. To this must be added the chronic bronchitis and frequent exacerbator phenotypes and the severity of airflow limitation [55,122], together with treatment with inhaled corticosteroids, which in a nationwide cohort of inhaled-corticosteroid users was followed by TB more often in patients with COPD than in those with asthma (odds ratio 2.31, 95% CI 1.39–3.38) [172]; all of these may modify local anti-mycobacterial defense. Where the phenotype studied is known, it is indicated below and in the summary tables; where the source does not specify it, that in itself is a limitation of the evidence.

### 9.3. Molecular Remodeling of Early Anti-Mycobacterial Defense

An important role here is played by a defect in early control involving the epithelial barrier and macrophages. Normally, mucus, cilia, and surface liquid remove a substantial fraction of inhaled particles before contact with phagocytes; in COPD, the mucus becomes viscous, the surface layer is dehydrated, ciliary function deteriorates, and mucociliary clearance declines [69,74], so that mycobacteria are retained in the airways for longer. Direct quantitative data on how much the dose of Mtb reaching the alveoli increases in COPD are insufficient, so this conclusion should be regarded as a biologically plausible assumption. Biochemical defense is also impaired: in a cell model, nicotine accelerates the intracellular growth of Mtb and reduces the production of β-defensins and LL-37 [73]. At the macrophage level in COPD, the cells become more numerous, but their functions suffer—phagocytosis, efferocytosis, and the resolution of inflammation are impaired [55]; the alveolar macrophages of patients are less able to take up apoptotic epithelial cells (an efferocytosis defect) [45] and bacteria, and azithromycin partially restores this process, indicating that part of the defect is reversible [46]. This signifies a shift in the macrophage “crossroads” toward permissiveness. The boundary of what is proven should, however, be maintained: direct studies of Mtb replication specifically in the macrophages of COPD patients are few, so the link between macrophage dysfunction and Mtb persistence remains a well-founded hypothesis.

A shift in lymphocyte composition is another mechanism. One of the most consistent features of COPD is the accumulation of CD8^+^ T cells in the peripheral airways, correlating with the degree of obstruction [94]; these Tc1-like cells are able to kill autologous lung cells, sustaining tissue destruction [95]. For TB, such cytotoxicity is ambiguous: it may remove infected cells, but in an already-damaged lung it more often intensifies destruction without a guaranteed improvement in control of Mtb replication. At the same time, the representation of early-response T cells declines—MAIT and iNKT cells [39] and, in the studies that assessed them directly, γδ T cells in induced sputum and bronchoalveolar lavage [149,150]—populations that are capable of partly compensating for the delay of the αβ response, whereas the role of B cells and of local lymphoid aggregates that sustain chronic inflammation increases [44].

One of the key mechanisms of tissue damage in COPD is neutrophilic inflammation, accompanied by a protease–antiprotease imbalance. Proteases, including MMP-12/macrophage elastase, contribute to extracellular matrix degradation and emphysema development [120]. An additional marker and potential participant in damage is the neutrophil extracellular trap (NET), which is detected in COPD and correlates with the degree of airflow limitation [121]. In the context of TB, this is of particular importance: neutrophils may participate in the early containment of infection, but on excessive activation they intensify necrosis, proteolytic damage, and destruction of lung tissue. γδ T cells are also potentially linked to this line of inflammation. In a model of emphysema, through IL-17A they stimulated the production of G-CSF and the generation of pathogenic Siglec-F^+^ neutrophils [111], which accords well with the notion of a dual role for the γδ T cell response. This mechanism cannot, however, be transferred directly to the combination of COPD and Mtb infection: it concerns an experimental model of emphysema rather than clinical COPD with TB in humans.

**Figure 3:**
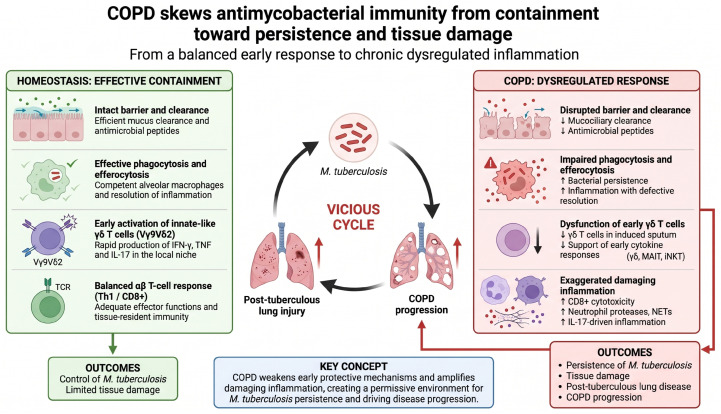
Proposed model of COPD-associated shifts in anti-mycobacterial immunity. The scheme summarizes a working model rather than an established causal sequence; the reduction in γδ T cells in induced sputum and bronchoalveolar lavage shown here is based on [149,150]. Black arrows indicate the proposed vicious cycle linking M. tuberculosis infection, post-tuberculosis lung injury, and COPD progression; green and red arrows indicate protective and detrimental pathways, respectively. ↑ and ↓ indicate increases and decreases, respectively. Created in BioRender. Oskin, D. (2026) https://BioRender.com/uk18e1f (accessed on 31 July 2026).

Thus, the data indicate a change in anti-TB defense in COPD (Figure 3; the individual components and the model in which each was observed are listed in Table 4). In COPD, the early mechanisms that should limit the entry and intracellular persistence of Mtb are weakened—mucociliary clearance, the antimicrobial activity of the epithelium, the phagocytic and resolving function of macrophages, and some of the rapid T cell populations. At the same time, the tissue-damaging reactions intensify—the cytotoxic CD8^+^ response, neutrophil proteases, NET formation, local lymphoid organization, and possibly IL-17-dependent neutrophilic inflammation. Reviews of the effects of smoking confirm that tobacco smoke disrupts both innate and adaptive defense mechanisms [47]. The association appears to be bidirectional: COPD is associated with an increased risk of active TB, and prior pulmonary TB is associated with subsequent chronic airflow obstruction; TB experienced in childhood and adolescence is associated with an increased risk of COPD and pre-COPD in adulthood ≤ 25 years. Outcomes were COPD and pre-COPD stages, including preserved ratio impaired spirometry (PRISm) and impaired spirometry (below Lower Limit of Normal (LLN) thresholds). Associations were evaluated using the multivariable logistic regression, adjusting for age, sex, body mass index, smoking, asthma, bronchiectasia, and air pollution. Among 10,178 patients (62 (9) years old) diagnosed with COPD, PTB during the developmental period was statistically associated with increased COPD risk across four models, showing a fully adjusted odds ratio (aOR) of 1.82 (95% CI: 1.45–2.26), and remained significant regardless of sex or smoking status. Critically, PTB was also independently associated with both PRISm and airflow obstruction (aOR  =  1.52, 95% CI: 1.35–1.71) and impaired spirometry below LLN (aOR  =  1.32, 95% CI: 1.14–1.52). Sensitivity analyses further reinforced the robustness of these findings: PTB during the developmental period is an independent risk factor for COPD and pre-COPD status in mid-to-late adulthood, necessitating long-term respiratory monitoring in PTB during the developmental period survivors and the implementation of targeted public health strategies [9]. This supports the notion of a vicious circle: chronic inflammation increases vulnerability to infection, while infectious damage intensifies tissue remodeling.

Consequently, COPD alters anti-mycobacterial defense at several levels at once, which makes the epidemiological link with an increased risk of TB biologically intelligible. At the same time, many of the links are proven only indirectly: direct studies of the COPD–γδ T cell–Mtb triad in humans are still few. The key task for future work is to determine which specific changes in lung tissue causally increase susceptibility to Mtb and which of them can be corrected without intensifying damaging inflammation.

### 9.4. High-Dimensional Immune Profiling: What It Has Added and What Is Still Missing

Much of what is now known about the spatial organization of the anti-mycobacterial response comes from methods that were unavailable when most of the studies cited above were performed. Multiplexed ion-beam imaging combined with transcriptomic analysis of human TB granulomas has shown that these lesions are not uniform structures but are organized into distinct microenvironments, with an IDO1- and PD-L1-expressing myeloid compartment forming an immunoregulatory zone that separates the lymphocyte cuff from the bacterial core [173]; single-cell sequencing has likewise resolved the coexisting macrophage states referred to in Section 3 rather than the binary M1/M2 scheme [52]. Spatial transcriptomic sequencing of human TB granulomas has since mapped the immune microenvironment of pulmonary lesions and shown that it differs by anatomical site [174], and paired single-cell and spatial profiling of human granulomas has identified an osteopontin-expressing macrophage population organized around the lesion core [175]. These approaches are directly relevant to the argument made here, because the central claim concerns *where* an effector cell acts and not only how many of them are present. What has not yet been done is the obvious combination: no study has applied paired single-cell transcriptomic, surface protein, and T cell receptor repertoire profiling together with spatial analysis to lung tissue from patients who have both COPD and TB. Until that is done, the γδ arm of the model proposed here remains inferential. Table 5 sets out, component by component, what is established in TB, what is established in COPD, what has been shown directly in the two together, and how strong the underlying evidence is.

## 10. Clinical and Therapeutic Implications

The mechanisms discussed above bear on clinical practice in two opposite directions: attempts to strengthen early anti-mycobacterial defense, and the consequences of deliberately suppressing it.

First, the practical conclusions are more cautious than the biology alone would suggest. Vaccine strategies that generate local airway immunity and trained myeloid reactivity are mechanistically attractive in COPD, where both are impaired, but no trial has shown that BCG or any other mycobacterial preparation modifies the course of COPD or prevents TB in this population, and the negative revaccination result discussed in Section 7 is a caution against extrapolating from immunogenicity to protection [165]. Immunomodulation directed at Vγ9Vδ2 cells—pAgs, their synthetic analogues, or aminobisphosphonates—has an established molecular rationale and preclinical support in primates [5,107,108], but its translation faces specific obstacles: there are no dose-finding data in chronic lung disease, the durability of an induced response is unknown, and the dual role of the γδ–IL-17 axis means that a technically successful expansion could aggravate neutrophilic inflammation in an already remodeled lung. Any clinical evaluation in TB–COPD would therefore need to be safety-gated on neutrophilic and proteolytic readouts, and not on IFN-γ alone.

### Targeted Immunomodulation in Patients with Latent or Active TB

The converse experiment is already being run in clinical practice. Biologic and targeted synthetic disease-modifying antirheumatic drugs (bDMARDs and tsDMARDs) interrupt individual cytokine pathways in defined ways, and the infection patterns that follow indicate which of the mechanisms described in this review are genuinely non-redundant in humans.

TNF inhibition is the clearest case: blockade of TNF reactivates latent infection, frequently early and with disseminated or extrapulmonary disease [92], confirming in humans what murine granuloma models had predicted about the role of TNF in maintaining granuloma integrity [91]. JAK inhibitors, which interrupt signaling downstream of IFN and several other cytokines, carry a lower but non-zero risk; in the tofacitinib clinical trial program the reported cases were concentrated in regions of high background TB incidence [177]. IL-17 inhibitors, by contrast, have not so far been associated with a clear excess of TB reactivation [178]—a pattern consistent with the picture developed here, in which IL-17 contributes more to neutrophil recruitment and tissue damage than to non-redundant control of Mtb. Concomitant glucocorticoids add independent, dose-related risk—current use was associated with an approximately fivefold higher odds of TB in a population-based case–control study, rising with the daily prednisone-equivalent dose [179]—and are easily overlooked when an event is attributed to the biologic agent.

Two distinctions govern management. The first is between latent TB infection, which is a matter of screening and preventive treatment, and active TB disease. Initiation of bDMARDs or tsDMARDs is generally deferred in active TB, whereas interruption and subsequent resumption of ongoing therapy should be determined according to the drug class, TB severity, antimicrobial susceptibility, and national guidance. The second distinction is between drug classes, since the magnitude of risk is clearly not uniform across them; decisions are best made jointly by the prescribing specialist and a TB physician, and we therefore do not propose fixed timings. What is consistent across guidance is the screening requirement: EULAR recommends screening for latent TB before bDMARDs and tsDMARDs, following national or international protocols, preferring an IGRA to the tuberculin skin test where available, and repeating screening when risk factors persist or arise [180]. In patients who also have COPD the problem is compounded, because the baseline defect in early anti-mycobacterial defense described above is added to the pharmacological one, and because we are not aware of studies validating IGRA performance specifically in chronic obstructive lung disease.

Read in the direction of this review, these agents are an informative natural experiment: suppressing one pathway at a time shows which arms of the early response are simultaneously protective and potentially injurious, and which can be interrupted with comparatively little cost to the control of Mtb.

## 11. Conclusions

A contemporary understanding of TB requires moving away from the model in which Mtb is regarded solely as an intramacrophage pathogen. The macrophage remains the first and key cell of the early interaction, but the outcome of infection is determined by the state of the local immune environment of the lung, where the epithelial barrier, alveolar and interstitial macrophages, αβ and γδ T cells, MAIT and iNKT cells, neutrophils, and structures of local lymphoid organization form a single system. The principal conclusion is that protection is determined not by the maximal intensity of inflammation but by its spatial and temporal organization: at the early stage, Mtb exploits the relative immunoregulatory orientation of alveolar macrophages, the disruption of phagosome maturation, epithelial routes of entry, and the delay of the αβ response, whereas later, control depends on whether the effector cells reach the appropriate regions of the tissue and on whether their activity turns into a source of immunopathology.

In this system, γδ T cells play a role that, while not central, is important. Their significance lies in their ability to connect early microbial and stress-induced recognition with the subsequent tissue T cell response. In humans and primates, pAg-reactive Vγ9Vδ2/Vγ2Vδ2 cells respond rapidly to mycobacterial metabolites through the BTN3A1/BTN2A1 axis, produce IFN-γ, TNF, and IL-17, enhance the activation of macrophages, and exhibit direct anti-mycobacterial activity; data from primate models and from BCG vaccination support the view that the targeted engagement of γδ T cells can enhance early control of infection. This does not, however, prove an independent or universal protective role for them—the function of γδ T cells depends on tissue context, subpopulation composition, the cytokine environment, and the stage of infection.

COPD substantially alters this context, disrupting mucociliary clearance, epithelial regeneration, the production of antimicrobial factors, the phagocytosis and efferocytosis of macrophages, and the balance between the protective response and chronic damaging inflammation. The increased susceptibility to TB in COPD is therefore probably linked not to a single defect but to a complex remodeling of the local response: at the early stage, Mtb more easily evades intracellular control, while the subsequent inflammation more often shifts toward tissue damage. With regard to γδ T cells, the most justified model appears to be not a simple one of “deficiency” or “activation” but one of functional reorientation: some components of the early response may be insufficient to control Mtb, whereas others sustain chronic immunopathology.

A key limitation of the existing data is that many mechanistic conclusions are based on a juxtaposition of different levels of evidence—studies in humans, primates, murine models, cell systems, and ex vivo observations. This is especially important for γδ T cells, because the human Vγ9Vδ2 system has no direct counterpart in standard mice, and part of the direct anti-mycobacterial activity is mediated by granulysin, which is likewise absent in ordinary mice. In addition, many of the links in the remodeling of immunity in COPD are proven only indirectly, and there are as yet no direct studies of the COPD–γδ T cell–Mtb triad in humans.

Further research should therefore be oriented not only toward a quantitative description of cells in the blood but also toward an analysis of local immunity in the airways and parenchyma: the study of tissue γδ subpopulations in COPD and TB, assessment of the “γδ–IL-17–neutrophil” axis, analysis of the interaction of γδ T cells with the epithelium and macrophages, and the search for biomarkers that distinguish a protective early response from inflammation that leads to damage. In practical terms, this means that TB in COPD should be regarded not only as an infectious complication but also as a manifestation of a disturbed local immune equilibrium. The most promising interventions—restoration of the barrier, correction of macrophage function, vaccine strategies that take tissue immunity into account, and cautious modulation of γδ- and IL-17-dependent mechanisms—are those that do not merely amplify inflammation but restore its proper spatiotemporal organization: sufficient to control Mtb yet not leading to progressive damage of lung tissue. Concretely, the questions raised here can be addressed by a defined program: longitudinal cohorts following patients with TB, with TB–COPD, with COPD alone and without lung disease from diagnosis through treatment and beyond; stratification of the COPD arms by etiology—tobacco, biomass, post-TB—and by phenotype rather than by FEV1 alone; paired sampling of blood, sputum, bronchoalveolar lavage and, where available, resected tissue; single-cell transcriptomic profiling with surface protein and T cell receptor repertoire readouts together with spatial profiling of the same specimens; functional rather than merely enumerative validation of the Vγ9Vδ2 and tissue non-Vδ2 subsets so identified; linkage of these immune measures to outcomes that matter clinically—cavitation, time to sputum conversion, relapse and the rate of post-treatment FEV1 decline; safety-gated early-phase trials of γδ-directed immunomodulation using neutrophilic and proteolytic readouts as stopping criteria; and separate study of patients receiving bDMARDs or tsDMARDs, in whom a single cytokine pathway is interrupted in a defined way and who therefore constitute an unusually informative natural experiment. Box 3 states the underlying open questions; the program set out above indicates how they could be answered.

Box 3 **Promising Directions for Research.**

*The COPD–TB–γδ T cell triad*

Determine the causal changes in lung tissue in COPD that increase susceptibility to Mtb and distinguish them from accompanying features.Characterize the effect of COPD on tissue non-Vδ2 and circulating Vγ9Vδ2 γδ T cells specifically in TB.Establish under what conditions the “γδ T cell–IL-17–neutrophil” axis performs a protective function and when it becomes a source of immunopathology.Develop vaccine and immunomodulatory approaches that induce an early local γδ response without enhancing neutrophilic damage.Evaluate the dynamics of Vγ9Vδ2 cells and of epithelial and macrophage markers as biomarkers of the protective versus the damaging early response.Study the influence of smoking, COPD, and post-TB remodeling on the antigen- and pAg-specific response, including the interpretation of IGRA.
*This box summarizes a research agenda for analyzing local immunity in the combination of COPD and TB.*

## Figures and Tables

**Table 1:** The cellular architecture of the early anti-TB response: protective function, pathological shift, and relevance in COPD.

Cell Population	Protective Function	Pathological Shift	Relevance in COPD	Key References
Alveolar macrophage	First uptake of Mtb; maintenance of alveolar homeostasis	With incomplete phagocytosis, early intracellular persistence; a “quiet,” anti-inflammatory profile facilitates the onset of infection	↑ number, but ↓ phagocytosis and efferocytosis; chronic activation	[3,15,16,17,18,45,46,47]
Interstitial/monocyte-derived macrophage	Antigen presentation, pro-inflammatory response	Higher Mtb replication; a site of proliferation and dissemination	Enhanced monocyte recruitment, dysregulation	[3,14,16,17,18,48,49,50,51,52,53,54,55]
Epithelium (AT2/AT1, club, basal cells)	Mucociliary clearance, surfactant, antimicrobial peptides, barrier regeneration	Mtb entry into AT2 and translocation; a source of DAMPs (damage-associated molecular patterns) upon injury	↓ clearance, ↓ β-defensins/LL-37, metaplasia, impaired repair	[4,19,20,21,22,23,24,56,57,58,59,60,61,62,63,64,65,66,67,68,69,70,71,72,73,74]
αβ T cells (CD4 Th1/Th17, CD8, TRM)	IFN-γ activation of macrophages, cytotoxicity, tissue memory	Delayed response; excess Th17/CD8 → tissue damage	Accumulation of cytotoxic CD8^+^ cells; shift toward damage	[2,25,26,27,75,76,77,78,79,80,81,82,83,84,85,86,87,88,89,90,91,92,93,94,95]
γδ T cells (Vγ9Vδ2; tissue non-Vδ2)	Rapid IFN-γ/TNF, granulysin-dependent activity, response to BCG	IL-17-dependent neutrophilic inflammation	Hypothesis: deficient early defense + IL-17 pathology	[5,28,29,30,31,32,96,97,98,99,100,101,102,103,104,105,106,107,108,109,110,111,112]
MAIT, iNKT, CD1-restricted cells	Rapid response to microbial metabolites and lipids	Reduction/dysfunction in chronic inflammation	↓ number and function	[33,34,35,36,37,38,39,113,114,115,116,117,118,119]
Neutrophils	Early control (ROS, granules, NETs)	Protease-mediated damage, necrosis, excessive NETs	A key damaging element (NE, MMP-12, NETs)	[40,41,104,105,111,120,121]
B cells/TLS (iBALT)	Organization of the local response	Maintenance of chronic inflammation	↑ lymphoid aggregates	[43,44,55,122]

**Note:** ↑ indicates an increase; ↓ indicates a decrease; → indicates a directional relationship or resulting effect.

**Table 2:** **γδ** T lymphocytes in TB: key aspects and level of evidence.

Aspect	Content	Level of Evidence	Context/Model	Key References
Subsets	Vγ9Vδ2 (blood, pAgs); Vδ1/non-Vδ2 (tissues, epithelium); murine IL-17^+^ γδ	Descriptive	Vγ9Vδ2—humans/primates; the mouse has different γδ cells	[31,96,97]
Recognition	Phosphoantigens (HMBPP/IPP) via BTN3A1 + BTN2A1	Well established	Humans/primates; molecular data	[28,29,32,98,99,100,101,102]
Effectors: cytokines	IFN-γ, TNF (macrophage activation)	Reasonably strong	Humans/primates	[30,103]
Effectors: cytotoxicity	Reduced Mtb viability through a granulysin-dependent mechanism	Reasonably strong	Humans, in vitro	[106]
IL-17	Early organization of the response vs. neutrophilic damage	Context-dependent	Mouse (Lockhart) + human (Peng)	[104,105]
Protection in vivo	Phosphoantigen/IL-2 expansion, immunization, and transfer of Vγ2Vδ2 control TB	Strong preclinical/non-human primate evidence	Non-human primates	[5,107,108]
BCG	Enhancement of the γδ response with features of memory; antibacterial functions of Vδ1/3	Moderately strong	Humans; some data recent/correlational	[109,110,112]
COPD	Deficiency (protection ↓) and IL-17-dependent pathology (harm); no direct “COPD + Mtb” data	Limited/hypothesis	Human (Pons; Urboniene; Szabó) + emphysema model (Hong)	[39,111,149,150]
Therapy in COPD–TB	Targeted modulation of the γδ axis	Hypothetical	No direct data	–

**Note:** ↓ indicates a decrease.

**Table 3:** BCG and the cellular immunity it generates.

Mechanism/Effect	Content	Context/Model	Key References
Clinical protection	High against disseminated forms in children; variable against adult pulmonary TB	Humans, meta-analyses of RCTs	[151,152]
Role of the route of administration	Intravenous BCG → pronounced protection; correlate—airway T cells	Macaques	[147,148]
Trained immunity (peripheral)	NOD2-dependent epigenetic/metabolic reprogramming of monocytes; NK memory	Humans, in vitro/ex vivo	[153,154,164]
Trained immunity (central level)	Reprogramming of hematopoietic progenitors	Mouse (Kaufmann) + human (Cirovic)	[155,156]
Heterologous protection	Reduced viremia in experimental viral infection	Humans, controlled infection	[157]
γδ component	Enhancement of the γδ response with memory traits; antibacterial functions of Vδ1/3	Humans; some data recent/correlational	[109,110,111,112]
Non-specific effects	↓ neonatal mortality; ↓ infections in the elderly; in adults—heterogeneous	Humans, RCTs	[158,159,160]
COPD	No direct data; association hypothetical, effect bidirectional	Open question	–
Vaccine strategies under evaluation	BCG revaccination; M72/AS01E; tissue/trained immunity	Humans, RCTs + review	[161,162,163]

**Note:** ↓ indicates a decrease; → indicates a directional relationship or resulting effect.

**Table 4:** Remodeling of anti-mycobacterial immunity in COPD.

Element	Change in COPD	Probable Consequence for Mtb Control	Context/Model	Key References
Barrier and clearance	↓ mucociliary clearance; ↓ antimicrobial peptides	↑ fraction of mycobacteria reaching the tissue	Humans + cell data	[69,73,74]
Alveolar macrophage	↓ phagocytosis/efferocytosis; dysregulation	Shift in the “crossroads” toward permissiveness	Humans, ex vivo	[45,46]
CD8^+^ T cells	↑ accumulation; cytotoxicity toward lung tissue cells	Tissue damage > protection	Humans	[94,95]
Innate-like cells	Deficiency of MAIT/iNKT/γδ	↓ early barrier defense	Humans	[39]
B cells/TLS	↑ role, lymphoid aggregates	Maintenance of chronic inflammation	Humans	[44]
Neutrophils and proteases	↑ NE, MMP-12, NETs	Matrix destruction; emphysema	Humans + mouse	[120,121]
γδ–IL-17 axis	IL-17A → G-CSF → pathogenic neutrophils	Immunopathology (duality of γδ)	Emphysema model	[111]
Integral risk	↑ susceptibility to TB; bidirectionality	The “COPD ↔ TB” loop	Meta-analyses, cohorts, review	[6,7,8,9,47]

**Note:** ↑ indicates an increase; ↓ indicates a decrease; → indicates a directional relationship or proposed pathway; ↔ indicates a bidirectional relationship.

**Table 5:** Immune alterations in TB, in COPD and in TB–COPD comorbidity, with the strength of the supporting evidence and the resulting clinical implication or evidence gap. “None” denotes the absence of published data obtained in patients with both diseases, not evidence of absence of an effect.

Immune Component	Evidence in TB	Evidence in COPD	Direct TB–COPD Evidence	Strength of Evidence	Clinical Implication/Gap
Epithelial barrier and clearance	Mtb enters AT2 cells; β-defensin-2 and LL-37 induced [4,24,63,64,65]	↓ clearance; ↓ antimicrobial peptides; metaplasia [69,73,74]	None; inferred from smoke and nicotine cell models [73]	Moderate; indirect	Delivered dose of Mtb in COPD unquantified
Alveolar macrophage	First intracellular niche; permissive early phenotype [3,15,16,17,18]	↓ phagocytosis and efferocytosis; partly reversible [45,46,55]	No study of Mtb growth in macrophages from COPD patients	Strong in each; none for overlap	Ex vivo infection of COPD alveolar macrophages
Autophagy and phagosome maturation	SapM and PknG block initiation and flux [124,125,126,136,137]	Autophagic flux impaired in alveolar macrophages of smokers, with defective delivery of bacteria to lysosomes [176]	None	Strong in each literature; not tested together in primary human alveolar macrophages	Additive effect of COPD untested
Vγ9Vδ2 T cells	pAg sensing via BTN3A1/BTN2A1; IFN-γ, TNF, granulysin; protection in macaques [5,28,29,100,101,102,106,107,108]	↓ γδ T cells in sputum and BAL; blunted smoking-associated expansion [149,150]; ↓ innate-like T cells overall [39]	None	Strong for sensing; primate-level for protection	Central gap: no phenotyping in TB–COPD
Tissue non-Vδ2 (Vδ1/Vδ3) cells	BCG induces antibacterial functions [112]	Not characterized	None	Recent, correlative	Requires tissue rather than blood
αβ CD4^+^ and CD8^+^ T cells	IL-12 → IFN-γ axis non-redundant; parenchymal CD4^+^ cells control Mtb [2,75,76,77,78,79,80,81,89]	↑ cytotoxic CD8^+^ cells in peripheral airways [94,95]	None with antigen-specific readouts	Strong in each; none for overlap	Cytotoxicity may injure without improving control
IL-17/neutrophil axis	γδ-derived IL-17 dominant early; ↑ IL-17^+^ γδ cells in active TB [104,105]	IL-17A → G-CSF → neutrophils; NE, MMP-12, NETs [111,120,121]	None; emphysema model only	Context-dependent; model-restricted	Key safety readout for γδ-directed therapy
B cells and tertiary lymphoid structures	Organise the local response [43,44]	↑ lymphoid aggregates sustaining inflammation [44,55,122]	None	Descriptive in both	Protective or injurious in the overlap is unclear
Trained immunity	NOD2-dependent reprogramming; HSPC-level training [153,155,156,164]	Not characterized; the myeloid compartment is already chronically activated [55,122]	None; revaccination did not prevent sustained IGRA conversion [165]	Mechanism strong; benefit unproven	Net benefit in a remodeled lung uncertain

**Note:** ↑ indicates an increase; ↓ indicates a decrease; → indicates a directional relationship or proposed pathway.

## Data Availability

The data are available from the corresponding author.

## Abbreviations

The following abbreviations are used in this manuscript:

A549	Human alveolar epithelial cell line
Ag85	Antigen 85 complex (Ag85A/B/C)
AT1/AT2	Alveolar epithelial type I/type II cell
B30.2	B30.2 (PRY/SPRY) intracellular domain of BTN3A1
BCG	Bacille Calmette–Guérin (*Mycobacterium bovis* BCG vaccine)
BTN2A1	Butyrophilin 2A1
BTN3A1	Butyrophilin 3A1
CARD9	Caspase recruitment domain-containing protein 9
CC16	Clara cell 16-kDa protein (secretoglobin SCGB1A1)
CD1	Cluster of differentiation 1 (lipid-antigen-presenting molecule)
CD4/CD8	Cluster of differentiation 4/8 T cell co-receptors
CFP-10	Culture filtrate protein, 10 kDa
cGAS	Cyclic GMP–AMP synthase
COPD	Chronic obstructive pulmonary disease
CXCR3	C-X-C chemokine receptor type 3
DAMP	Damage-associated molecular pattern
DC-SIGN	Dendritic cell-specific ICAM-3-grabbing non-integrin (CD209)
Dectin-2	Dendritic cell-associated C-type lectin 2
ESAT-6	Early secreted antigenic target, 6 kDa
ESX-1	ESAT-6 secretion system 1 (type VII secretion system)
Foxp3	Forkhead box P3
G-CSF	Granulocyte colony-stimulating factor
GSDMD	Gasdermin D
HIV	Human immunodeficiency virus
HLA	Human leukocyte antigen
HMBPP	(E)-4-hydroxy-3-methylbut-2-enyl pyrophosphate
iBALT	Inducible bronchus-associated lymphoid tissue
IFN-γ	Interferon-γ
IGRA	Interferon-γ release assay
IL	Interleukin (e.g., IL-1β, IL-2, IL-12, IL-17, IL-17A, IL-23)
iNKT	Invariant natural killer T cell
IPP	Isopentenyl pyrophosphate
KLRG1	Killer cell lectin-like receptor G1
LAM	Lipoarabinomannan
LL-37	Human cathelicidin antimicrobial peptide LL-37
MAIT	Mucosal-associated invariant T cell
ManLAM	Mannose-capped lipoarabinomannan
MCL	Macrophage C-type lectin (CLEC4D)
MEP	2-C-methyl-D-erythritol 4-phosphate (non-mevalonate) pathway
MHC	Major histocompatibility complex
Mincle	Macrophage-inducible C-type lectin (CLEC4E)
MLKL	Mixed-lineage kinase domain-like protein
MMP-12	Matrix metalloproteinase-12 (macrophage metalloelastase)
MSMD	Mendelian susceptibility to mycobacterial disease
Mtb	*Mycobacterium tuberculosis*
MUC5AC/MUC5B	Mucin 5AC/mucin 5B
MVA85A	Modified vaccinia Ankara virus expressing antigen 85A
NE	Neutrophil elastase
NET	Neutrophil extracellular trap
NK	Natural killer cell
NLRP3	NOD-, LRR-, and pyrin domain-containing protein 3
NOD2	Nucleotide-binding oligomerization domain-containing protein 2
PD-1	Programmed cell death protein 1
pAg	Phosphoantigen
PPD	Purified protein derivative (tuberculin)
PtpA	Protein tyrosine phosphatase A
RD1	Region of difference 1
RIPK1/RIPK3	Receptor-interacting serine/threonine protein kinase 1/3
ROS	Reactive oxygen species
SapM	Secreted acid phosphatase M
SP-A/SP-D	Surfactant protein A/D
STING	Stimulator of interferon genes
Syk	Spleen tyrosine kinase
TB	Tuberculosis
Tc1	Type 1 cytotoxic T cell
TCR	T cell receptor
TDM	Trehalose dimycolate (cord factor)
Th1/Th17	T helper type 1/type 17 cell
TLRs	Toll-like receptors
TLR2	Toll-like receptor 2
TLS	Tertiary lymphoid structure
TNF	Tumor necrosis factor
Treg	Regulatory T cell
TRM	Tissue-resident memory T cell
Vγ9Vδ2/Vγ2Vδ2	γδ T cell receptor usage defining the major pAg-responsive subset (human Vγ9Vδ2; non-human primate Vγ2Vδ2)
Vδ1/Vδ3	γδ T cells defined by δ-chain (Vδ1, Vδ3) usage
αβ T cell	T cell bearing an α/β T cell receptor
γδ T cell	T cell bearing a γ/δ T cell receptor
